# Plasticity of Performance Curves in Ectotherms: Individual Variation Modulates Population Responses to Environmental Change

**DOI:** 10.3389/fphys.2021.733305

**Published:** 2021-09-28

**Authors:** Frank Seebacher, Alexander G. Little

**Affiliations:** ^1^ School of Life and Environmental Sciences, University of Sydney, Sydney, NSW, Australia; ^2^ Department of Biology, Biosciences Complex, Queen’s University, Kingston, ON, Canada

**Keywords:** bet-hedging, plasticity, swimming performance, acclimation, environmental variation

## Abstract

Many ectothermic animals can respond to changes in their environment by altering the sensitivities of physiological rates, given sufficient time to do so. In other words, thermal acclimation and developmental plasticity can shift thermal performance curves so that performance may be completely or partially buffered against the effects of environmental temperature changes. Plastic responses can thereby increase the resilience to temperature change. However, there may be pronounced differences between individuals in their capacity for plasticity, and these differences are not necessarily reflected in population means. In a bet-hedging strategy, only a subsection of the population may persist under environmental conditions that favour either plasticity or fixed phenotypes. Thus, experimental approaches that measure means across individuals can not necessarily predict population responses to temperature change. Here, we collated published data of 608 mosquitofish (*Gambusia holbrooki*) each acclimated twice, to a cool and a warm temperature in random order, to model how diversity in individual capacity for plasticity can affect populations under different temperature regimes. The persistence of both plastic and fixed phenotypes indicates that on average, neither phenotype is selectively more advantageous. Fish with low acclimation capacity had greater maximal swimming performance in warm conditions, but their performance decreased to a greater extent with decreasing temperature in variable environments. In contrast, the performance of fish with high acclimation capacity decreased to a lesser extent with a decrease in temperature. Hence, even though fish with low acclimation capacity had greater maximal performance, high acclimation capacity may be advantageous when ecologically relevant behaviour requires submaximal locomotor performance. Trade-offs, developmental effects and the advantages of plastic phenotypes together are likely to explain the observed population variation.

## Introduction

Temperature is one of the most relevant physical state variables in biology because physiological rates and hence fitness are influenced by the thermal environment (Tattersall et al., 2012). High temperatures, in particular, cause damage to proteins and membranes and can thereby disrupt fundamental processes such as movement, growth and reproduction (Tattersall et al., 2012). Variation in the thermal environment can be a strong predictor of individual fitness and population persistence (Kingsolver and Buckley, 2017). In the current era of global warming, understanding thermal effects on organisms has assumed a new urgency because of their potential role in determining the success and biogeography of populations and species (Sinclair et al., 2016; Woods et al., 2018).

At a reductionist level, living organisms are comprised of networks of interacting biochemical pathways (Costanzo et al., 2021). Thermodynamics dictates that the rate of biochemical reactions depends on the temperature of the system. The thermal sensitivity of higher organismal traits such as locomotor performance or metabolic rate is then determined by the thermodynamics of flux through underlying biochemical pathways. Hence, each physiological rate has a characteristic temperature response, which is captured by ‘thermal performance curves’ (TPC; Huey and Kingsolver, 1989).

**Figure 1:**
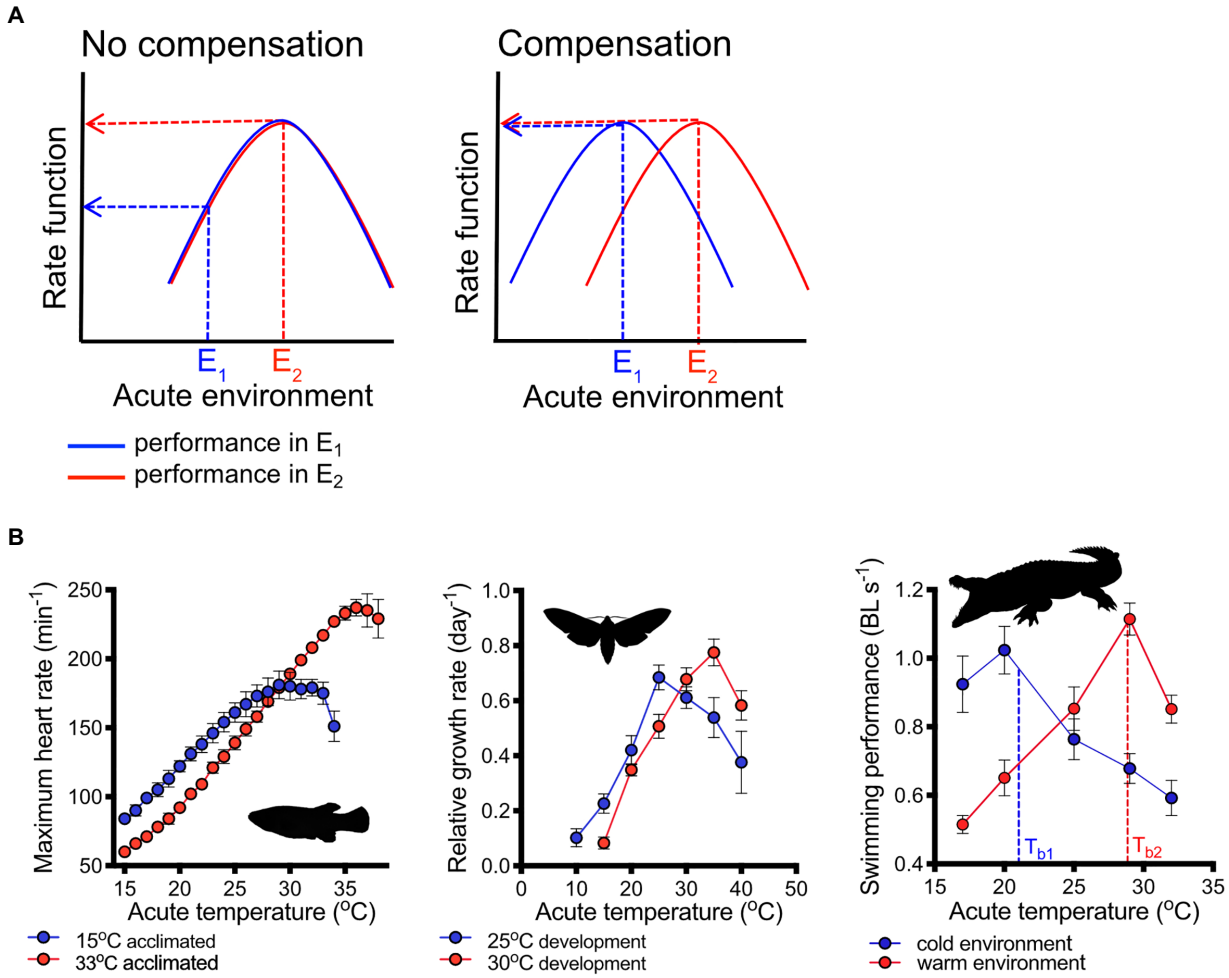
Plasticity of thermal performance curves. Ideally, plasticity can shift performance curves so that physiological rate functions are perfectly buffered from changes in the thermal environment. The case of perfect compensation is shown (**A**, right panel) schematically as a right shift of the thermal performance curve (blue line=performance in cool environment, E1; red line=performance in warm environment, E2). Lack of compensation (left panel) would result in a substantial decrement in performance as temperatures decrease. Examples **(B)** of shifts in performance curves demonstrating different degrees of compensation: heart rate in killifish [*Fundulus heteroclitus*; redrawn from Safi et al. (2019), left panel] and growth rates in moth (*Maduca sexta*) larvae [central panel, redrawn from Kingsolver et al. (2020)] compensate partially for different acclimation or developmental temperatures, respectively. Thermal performance curves of swimming in crocodiles (*Crocodylus porosus*, right panel) thermoregulating in simulated winter (cool) and summer (warm) environments shifted horizontally so that swimming performance remained nearly constant at the different regulated body temperatures [T_b1_ and T_b2_ in cool and warm environments, respectively; redrawn from Glanville and Seebacher (2006)]. Means ± s.e. are shown in **(B)**, and images are from PhyloPic (http://phylopic.org/).

A TPC describes the change in a physiological rate across a range of acutely changing temperatures. The shape of TPCs is characteristically in the form of an inverted ‘U’, where rates increase with an increase in temperature until a maximum is reached beyond which rates decline with further temperature increases (Huey and Kingsolver, 1989; McKenzie et al., 2021; Figure 1). The decrease in rates at lower temperatures is caused by thermodynamic constraints in Gibb’s free energy, while the decline in rates at high temperatures results from a loss of the quaternary or tertiary structures of enzymes and damage to membranes (DeLong et al., 2017).

However, TPCs are not fixed within individuals over time (Sinclair et al., 2016) or consistent among individuals within populations or species (Careau et al., 2014; Seebacher et al., 2015). Long-term exposure to different temperature regimes within or across generations can shift the TPC of individuals. Hence, transgenerational, developmental or reversible plasticity can result in changes in the maximum, mode and breadth of TPCs (Schulte et al., 2011; LeRoy et al., 2017). These epigenetic effects are at least partly regulated by DNA methylation and histone acetylation (Simmonds and Seebacher, 2017; Loughland et al., 2021). Plasticity can be beneficial if performance is optimised at the acute thermal environment experienced by individuals. However, there is a potential cost to plasticity if the temperature range at which performance maxima occur mismatches the prevailing thermal conditions in the environment (Bateson et al., 2014).

Importantly, TPCs and their plasticity also vary between individuals within populations (Seebacher et al., 2015). A potential ramification of this individual variation is that means of thermal performance across samples of individuals do not necessarily represent the population as a whole. Instead, population responses may be determined by a bet-hedging strategy (Haaland et al., 2020). In a bet-hedging scenario, populations comprise individuals with high capacity for acclimation that can fully compensate for an environmental change given sufficient time to acclimate and individuals with low acclimation capacity that cannot compensate physiological rates at all. We found this to be the case in mosquitofish (*Gambusia holbrooki*; Seebacher et al., 2015; Loughland and Seebacher, 2020). It is possible that variation in the plasticity of TPCs may disadvantage some individuals under particular conditions, but promote population resilience as a result of the increased diversity of phenotypes (Schindler et al., 2015). Our aim was to explore these relationships to document variation in the plasticity of TPCs between individuals and to test how this variation may affect population responses. In mosquitofish, we also observed a trade-off between the capacity for acclimation and performance under warm conditions (Seebacher et al., 2015). Hence, here, we model how differences in the plasticity of TPCs together with this trade-off affect population performance in different thermal environments.

We collated a large data set from previously published studies on swimming performance of mosquitofish (608 individuals), in which each individual was acclimated twice to determine the capacity for reversible plasticity (Seebacher et al., 2015; Loughland and Seebacher, 2020). We subsampled this data set to characterise the effects of variation in individual phenotypes on population responses. This data set is unique because data from double-acclimated animals that permit calculation of individual plasticity are rare and because the large number of samples available makes our modelling approach possible; to the best of our knowledge, similar data sets do not exist for other species. However, we acknowledge that data from a single species may lack generality, and our results represent a proof of concept but do not necessarily apply to all ectotherms. Our aims were (a) to characterise the variation in plasticity of TPCs between individuals within populations and (b) to determine the extent to which sample means drawn from population mask individual variation. Finally, (c) we modelled the extent to which variation in capacity for plasticity between individuals influences population responses to environmental temperature variation.

## Materials and Methods

### Data

We re-analysed three different data sets on thermal acclimation of swimming performance (critical sustained swimming speed, U_crit_) in mosquitofish (*Gambusia holbrooki*; Seebacher et al., 2015; Loughland and Seebacher, 2020). In each data set, individual fish were acclimated twice, to a cool (18 or 20°C) and to a warm (28°C) temperature in random order; acclimation temperatures corresponded to spring and summer temperatures at the capture site (Seebacher et al., 2014). All fish were sourced from the same wild population (Manly Dam, Australia 33°78′S; 151°26′E) and were acclimated for 3–4weeks to the different temperatures. After acclimation, U_crit_ of each fish was measured at different acute test temperatures. Experimental fish were of mixed sex (184 females, 424 males) and were sexually immature at the start of acclimation treatments. Fish were separated from each other during acclimation treatments to ensure that females were not pregnant at the time of U_crit_ measurements.

In the first data set (data set 1), *n*=48 wild-caught fish were each acclimated to 20 and 28°C in random order, and after each acclimation treatment, U_crit_ was measured at 20, 28 and 32°C acute test temperatures in each fish (Seebacher et al., 2015). In the second data set (data set 2), U_crit_ of 416 double-acclimated (to 18 and 28°C), wild-caught fish was measured only at the acute test temperature that coincided with acclimation temperatures (Loughland and Seebacher, 2020). The third data set (data set 3) from the same publication (Loughland and Seebacher, 2020) was collected from third- or fourth-generation offspring (total *n*=144 fish) bred in outdoor mesocosms from parents collected at the study site. Each of these fish was acclimated to 20 and 28°C, and U_crit_ of each fish was measured at 20 and 28°C acute test temperatures after each acclimation treatment.

Double acclimation permitted calculation of an index of acclimation capacity for each fish (Seebacher et al., 2015):

$$\mathrm{Acclimation capacity}=1-\frac{\left(P_{28}-P_{20}\right)}{\left[\left(P_{28}+P_{20}\right)/2\right]},$$

where P_28_ is the U_crit_ of a fish that is acclimated to 28°C and measured at 28°C acute test temperature, while P_20_ is the equivalent measure at 20°C (or 18°C in data set 2). The acclimation capacity index indicates relative thermal compensation (i.e. the ability to maintain relatively constant performance across thermal conditions) by contrasting the difference between P_20_ and P_28_. Acclimation capacity approaches 1 as P_20_ approaches P_28_ and decreases as P_20_ decreases. If a fish overcompensated for low temperatures and P_20_>P_28_, the index will be >1. The index is based on the difference between P_20_ and P_28_ and is a dimensionless number that is independent from the absolute values of P_20_ and P_28_. More details of experimental procedures are given in the original publications (Seebacher et al., 2015; Loughland and Seebacher, 2020).

### Consequences of Variation on Interpretation of Samples

Our purpose here was to mimic typical approaches in the literature to assess acclimation of populations to test whether subsampling of populations can reflect true variation and responses of the population. Hence, we randomly subsampled the combined data sets 1 and 3 (*n*=192 fish, see Supplementary Material for R code) to draw 10 samples of eight replicates each for warm- and cold-acclimated fish. Each of these 10 data sets mimics a fairly typical experiment in the literature, for example as in our study on mosquitofish where we compared acclimation in spring- and summer-caught fish (Seebacher et al., 2014).

We analysed each data set with a permutational analysis [in the R package lmPerm (Wheeler and Torchiano, 2016)] with acclimation temperature and acute test temperature as independent factors. Permutational analyses do not make assumptions about underlying data distributions but use the data *per se* to infer significant differences (Drummond and Vowler, 2012). Hence, values of *p* in permutational analyses are not associated with any particular distribution, and there are no test statistics (such as F or t; Ludbrook and Dudley, 1998). The value of *p* in permutational tests has the practical meaning of denoting the number of randomised permuted data sets for which the treatment effects were as or more extreme than the observed experimental data divided by the total number of permutations.

### Consequences of Individual Variation for Population Responses

Our aim here was to model how populations comprised of individuals with different acclimation capacities respond to different environmental conditions. We modelled different phenotypic compositions of populations by selecting subpopulations from the complete data set (data sets 1, 2 and 3 combined; *n*=608 fish), which were the top 10% of fish with the highest acclimation capacity (high), the bottom 10% with the lowest acclimation capacity (low) and the central 10% (centre); each of these subpopulations was comprised of 61 fish.

From the P_20_ and P_28_ values of each fish, we determined the slope of change in U_crit_ between these temperatures to estimate U_crit_ at intermediate temperatures assuming that acclimation has taken place. We simulated environmental conditions within the measured range (20–28°C) by either assuming constant conditions of 20 or 28°C or letting temperatures vary between 20 and 28°C. To model variable temperatures, we assumed sinusoidal temperature variation with a mean of 24°C and an amplitude of 4°C. We randomised the phase of the sinusoidal temperature fluctuation 100 times for each fish and recalculated U_crit_ (from the slope between P_20_ and P_28_) each time. We thereby ‘exposed’ each fish to the complete temperature variation. From the simulated data set, we calculated mean U_crit_ and 95% confidence intervals for each subpopulation (using *n*=61 for CI calculations to represent the number of fish in the simulation rather than the number of simulated values).

Mean values for each subpopulation may mask the underlying distributions of U_crit_, which may be important for ecological responses. For each subpopulation (low, centre, high acclimation capacity), we therefore modelled U_crit_ distributions in the variable environment (as determined above) as the per cent of U_crit_ values that fell above a given fraction of maximum speed. The low acclimation capacity subpopulation had the highest maximum U_crit_, which we used as maximum U_crit_ in all simulations. However, rather than using the single highest U_crit_ value, which is not representative of most fish, we defined the 90th percentile of the low acclimation capacity subpopulation as the maximum U_crit_. We then determined the percentage of U_crit_ values that fell above a given fraction of this maximum U_crit_ for each subpopulation, which we defined as ‘achievable’ U_crit_. See Supplementary Material for R code.

## Results and Discussion

### Variation in Plasticity of Thermal Performance Curves Between Individuals

**Figure 2:**
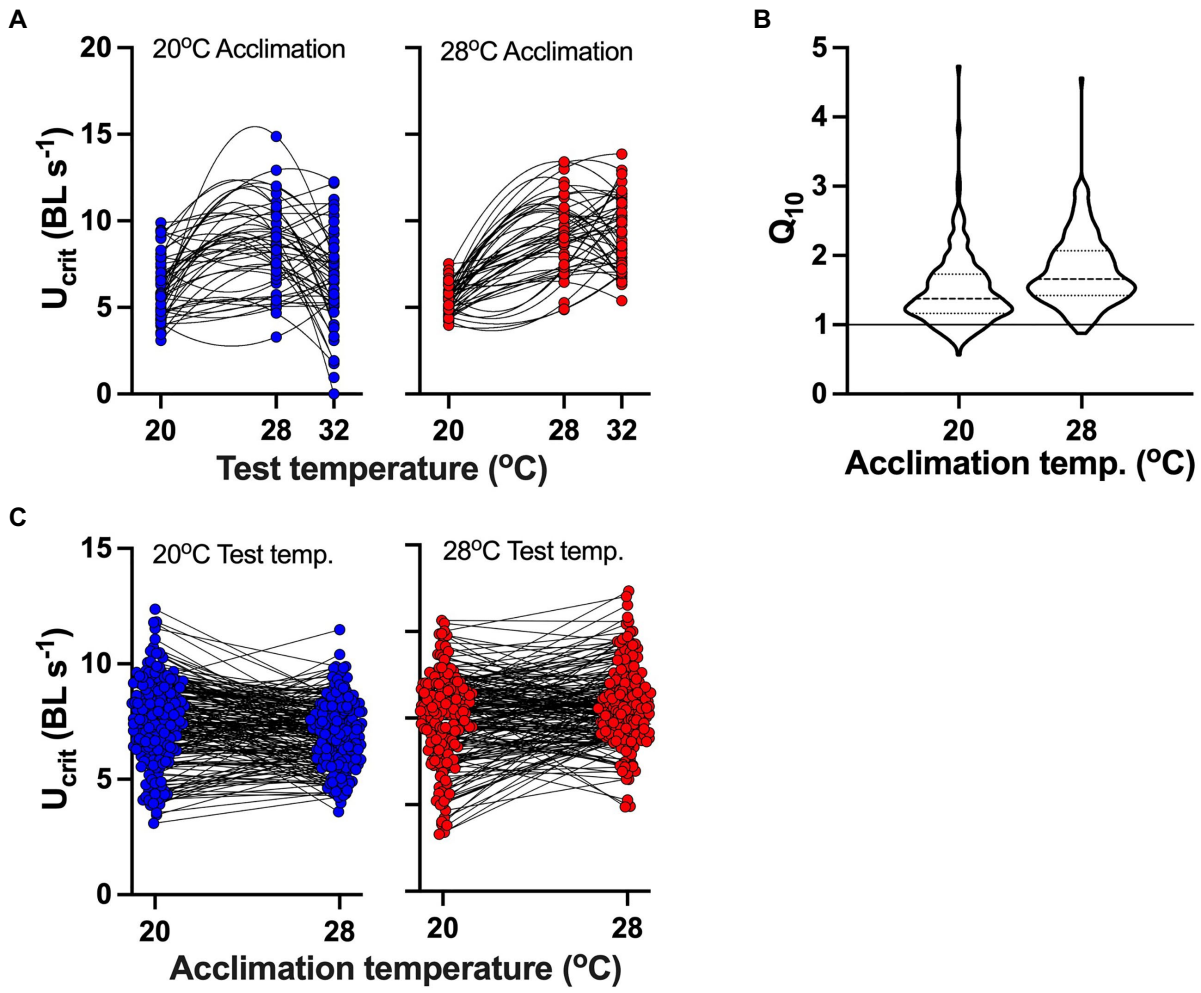
Variation in individual responses to different acclimation and test temperatures. **(A)** Nonlinear performance (quadratic fit) curves for a subset (*N*=48; data set 1) of fish for which we recorded swimming performance at three test temperatures; blue symbols indicate acclimation to 20°C, and red symbols indicate acclimation to 28°C. **(B)** Violin plot of thermal sensitivity (Q_10_ values between 20 and 28°C acute test temperatures) of individuals (from data sets 1 and 3; *n*=192 fish) shows considerable variation among individuals acclimated to 20 and 28°C. Thick broken line in violin plots shows mean, and thin broken lines show 95% confidence intervals; the solid line in the plot indicated Q_10_=1, below which U_crit_ decreased with increasing test temperature. **(C)** Change in performance across acute test temperatures following acclimation to different temperatures. Thin black lines connect datapoints taken from the same individual. Note the pronounced differences in the directions of change in response to different acute test and acclimation temperatures. **(B,C)** are plots of the same data.

Thermal performance curves varied considerably between individuals (Figure 2). In both cold- and warm-acclimated fish from data set 1 (Figure 2A), U_crit_ tended to increase from 20 to 28°C and decrease at 32°C. However, this pattern was not consistent among individuals, and frequently U_crit_ increased between 28 and 32°C. In fish from data sets 1 and 3 (*n*=192; Figure 2B), U_crit_ mostly increased between 20 and 28°C at both acclimation temperatures, but there was considerable variation in thermal sensitivity (i.e. Q_10_ values). Similarly, responses to acclimation differed considerably between individuals (Figure 2C). At both 20 and 28°C test temperatures, acclimation to 28°C led to either increased or decreased performance in different individuals (data from data sets 1 and 3).

**Figure 3:**
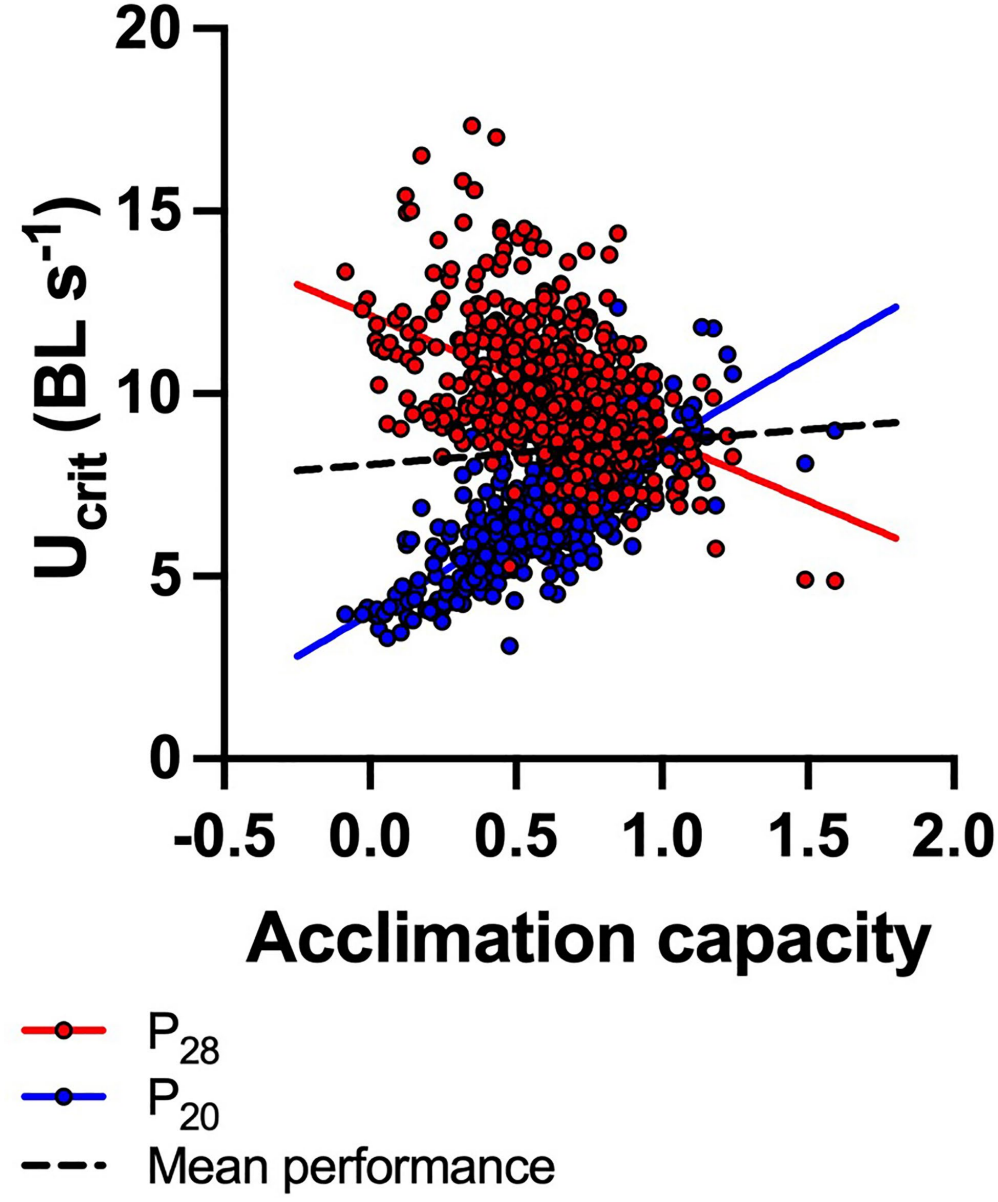
Trade-off between capacity for acclimation and swimming performance (U_crit_) under warm conditions. Acclimation capacity is shown as a dimensionless index (see main text) that indicates the capacity for reversible acclimation (1=perfect compensation; >1=cold-acclimated fish perform better; <1=warm-acclimated fish perform better). Performance at 28°C test temperature of fish acclimated to 28°C (P_28_, red circles) decreased as acclimation capacity increased, while P_20_ (U_crit_ of 20°C acclimated fish measured at 20°C test temperature) increased. Across both acclimation treatments, mean performance increased slightly with increasing acclimation capacity (broken black line). *N*=608 fish (both P_20_ and P_28_ are plotted for each fish), and significant regression lines are shown (P_20_: Y=3.97+4.67x; *R*^2^=0.54, *p*<0.0001; P_28_: Y=12.15−3.39x; *R*^2^=0.22, *p*<0.0001; mean performance: Y=8.06+0.68x, *R*^2^=0.014, *p*=0.0032).

Similar to the patterns of thermal sensitivity described above, there was pronounced variation in acclimation capacity within the total population (data sets 1–3, *n*=608 fish; Figure 3). Fish phenotypes ranged from having the capacity to fully compensate (and even overcompensate) for the 8–10°C temperature difference following acclimation, to having no capacity for acclimation at all so that U_crit_ changed passively (thermodynamically) with an acute change in temperature. Interestingly, there was a trade-off in capacity for acclimation with performance at warm conditions (P_28_): P_28_ decreased significantly (regression: Y=12.15−3.39x; *R*^2^=0.22, *p*<0.0001) as P_20_ increased (Y=3.97+4.67x; *R*^2^=0.54, *p*<0.0001) with increasing acclimation capacity. Across both acclimation treatments, mean performance of fish increased slightly but significantly with increasing acclimation capacity (Y=8.06+0.68x, *R*^2^=0.014, *p*=0.0032; Figure 3). However, the persistence of both plastic and fixed phenotypes in the population indicates that on average, plasticity is not necessarily advantageous over fixed phenotypes, but also that plasticity does not carry a cost that would select against it.

Increased variation of phenotypes can render the populations as a whole more resilient to change if different phenotypes are advantageous under different environmental conditions (Schindler et al., 2015; Blondel et al., 2021). Plasticity is advantageous by buffering performance under cooler conditions, such as in winter, while fixed phenotypes perform better at high temperatures during summer. This evolutionary bet-hedging can increase population persistence (Schindler et al., 2015). Mechanistically, it is an interesting question of what mediates the observed variation between individuals. Reactive oxygen species (ROS) and heat shock proteins can be induced by acute heat or cold exposure (Liu et al., 2018). Cold acclimation also increased ROS production in mosquitofish (Loughland and Seebacher, 2020) and grass snakes (Bury et al., 2018), and salmon acclimated to 20°C had greater rates of oxidative phosphorylation but reduced ROS production compared to 12°C acclimated fish (Gerber et al., 2020). Mosquitofish with high acclimation capacity also have greater antioxidant capacities (Loughland and Seebacher, 2020). Increased ROS as a result of reduced antioxidant capacity can decrease swimming performance (Ghanizadeh-Kazerouni et al., 2016) and may at least partly explain the observed patterns in our study. Alternatively, the dynamics of signalling pathways, from endocrine (e.g. thyroid hormone) to epigenetic (e.g. histone acetylation and DNA methylation), may differ between individuals and thereby cause individual variation (Simmonds and Seebacher, 2017; Little, 2021; Loughland et al., 2021).

### Consequences of Individual Variation on Interpretation of Samples

**Figure 4:**
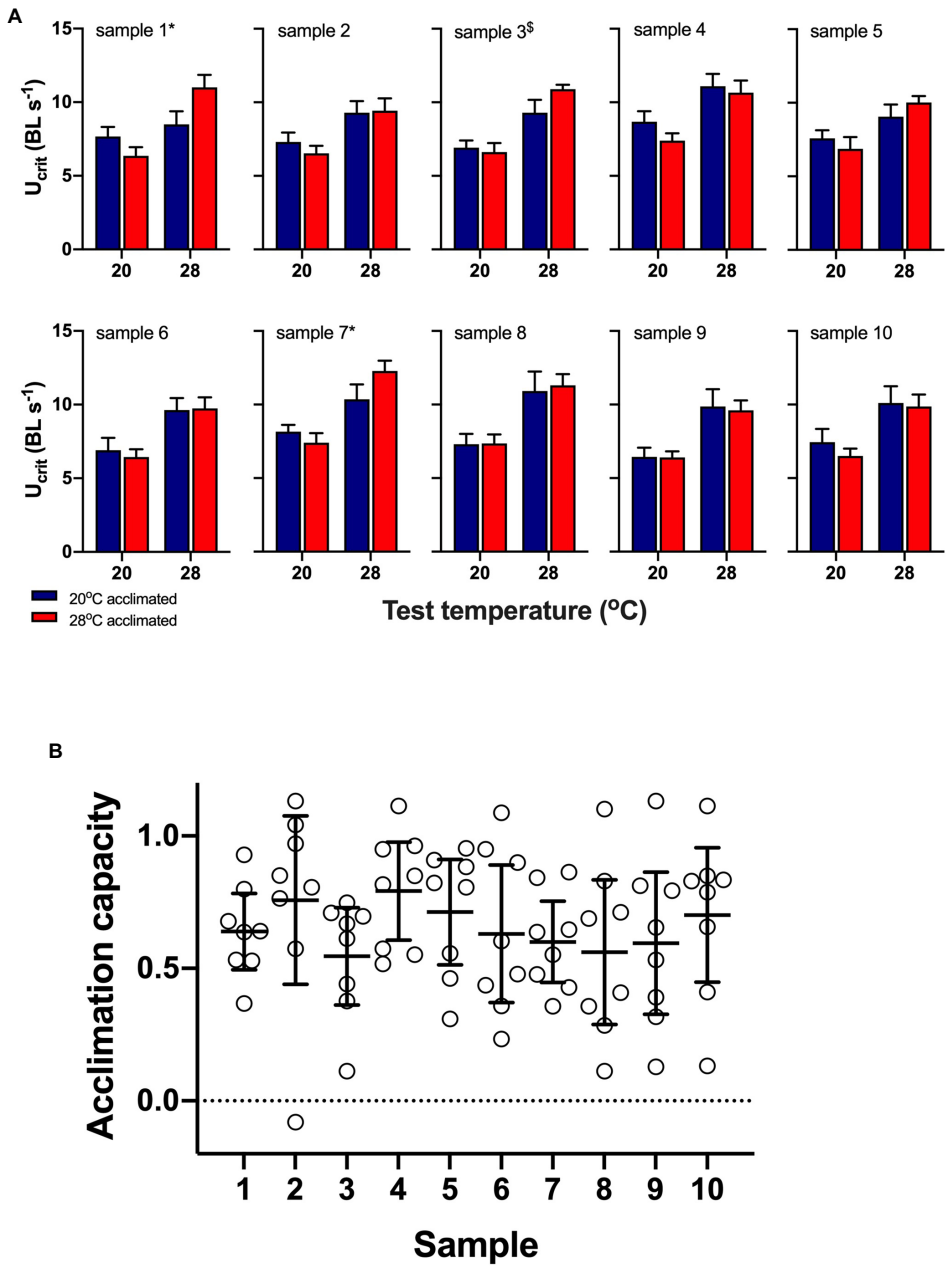
Data from ten random samples of eight fish from the pool of 192 fish (data sets 1 and 3). **(A)** mean (± s.e.) data from the ten samples showing swimming performance (U_crit_) of 20°C (blue bars) and 28°C (red bars) acclimated fish measured at 20 and 28°C acute test temperatures. Two-factor permutational analyses showed that acclimation did not have a significant main effect in any sample, but test temperature was significant in all samples. There was a significant interaction between test and acclimation temperatures in samples 1 and 7 (*p*=0.013 and *p*=0.044, respectively, indicated by ^*^; sample 3: *p*=0.083 indicated by ^$^). **(B)** Means (± s.e.) mask the variation in acclimation capacity between individuals, and in most samples, individual acclimation capacity ranged from close to no capacity for acclimation (values→0) to perfect compensation for the acclimation temperature difference between treatments (values→1).

Acclimation did not have a significant main effect in any of the ten random samples (all *p*>0.3), but test temperature was significant in all samples, and on average, U_crit_ increased with increasing test temperature (all *p*<0.003; Figure 4A). There was a significant interaction between test and acclimation temperatures in samples 1 and 7 (*p*<0.05) and at a one-tailed significance level in sample 3 (*p*=0.083); the interaction was not significant in any of the other samples (all *p*>0.24). Knowing the true acclimation capacity of individual fish showed that means mask the variation in acclimation capacity between individuals, and in many samples, individual acclimation capacity ranged from close to no capacity for acclimation (values→0) to perfect compensation for the acclimation temperature difference between treatments (values→1; Figure 4B).

The relatively small sample size simulated here can lead to fundamentally different conclusions about the population as a whole: in most cases, the data showed that there was no acclimation response, but three samples indicated that there was. These results serve as a cautionary note to avoid undersampling of populations and presenting means in the absence of individual values. However, even if larger samples were collected, sample means would mask the underlying variation and obscure the bet-hedging dynamics discussed above. Experimental approaches that compare individuals from different acclimation treatments may not be sufficient to test for costs or trade-offs associated with plasticity, which would require knowledge of within-individual acclimation capacities. Understanding individual variation in acclimation capacity would be necessary to predict how populations can respond to climate variability, where diversity of phenotypes may be important to increase resilience. Sample means can show population trends across time or contexts.

### Consequences of Variation for Population Responses

**Figure 5:**
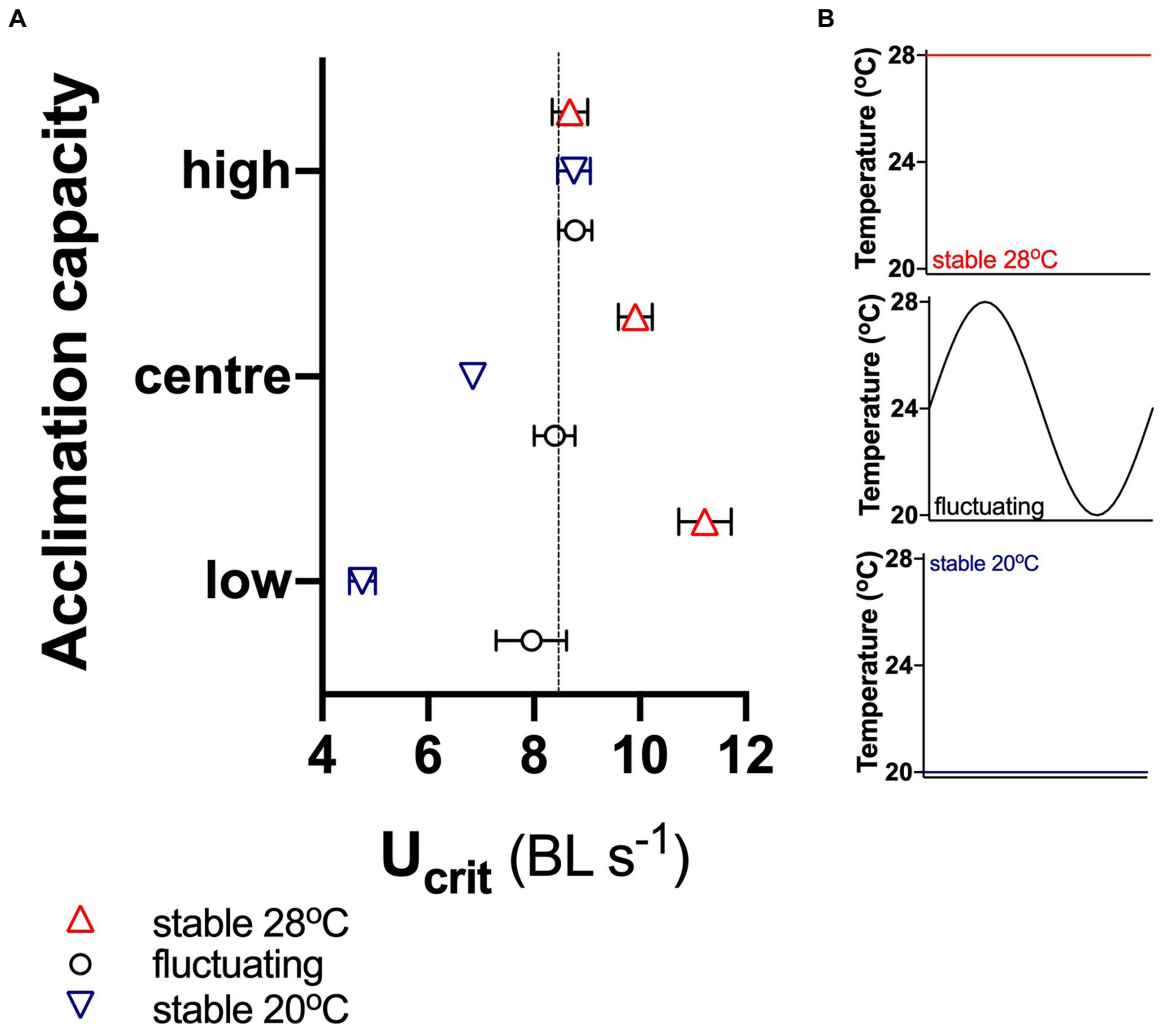
Simulated responses to environmental change. **(A)** U_crit_ of fish with high acclimation capacity (top 10% of the total population of 608 fish), with low acclimation capacity (bottom 10%) and of the central 10% of the total population in different environments: stable at 20 and 28°C and fluctuating between 20 and 28°C **(B)**. Means ± 95% confidence intervals are shown, and the dotted horizontal line in **(A)** indicates the mean of the total population of 608 fish in the fluctuating environment.

Our simulations showed that in the fluctuating environment, the average U_crit_ was similar in all three subpopulations, indicating that capacity for acclimation did not affect mean performance under these circumstances (Figure 5). However, performance in the stable cool environment decreased in the centre and low acclimation subpopulations, but it increased in the stable warm environment in those subpopulations. In contrast, U_crit_ of fish with high acclimation capacity did not vary significantly (95% CI) between either the stable or the fluctuating environments.

**Figure 6:**
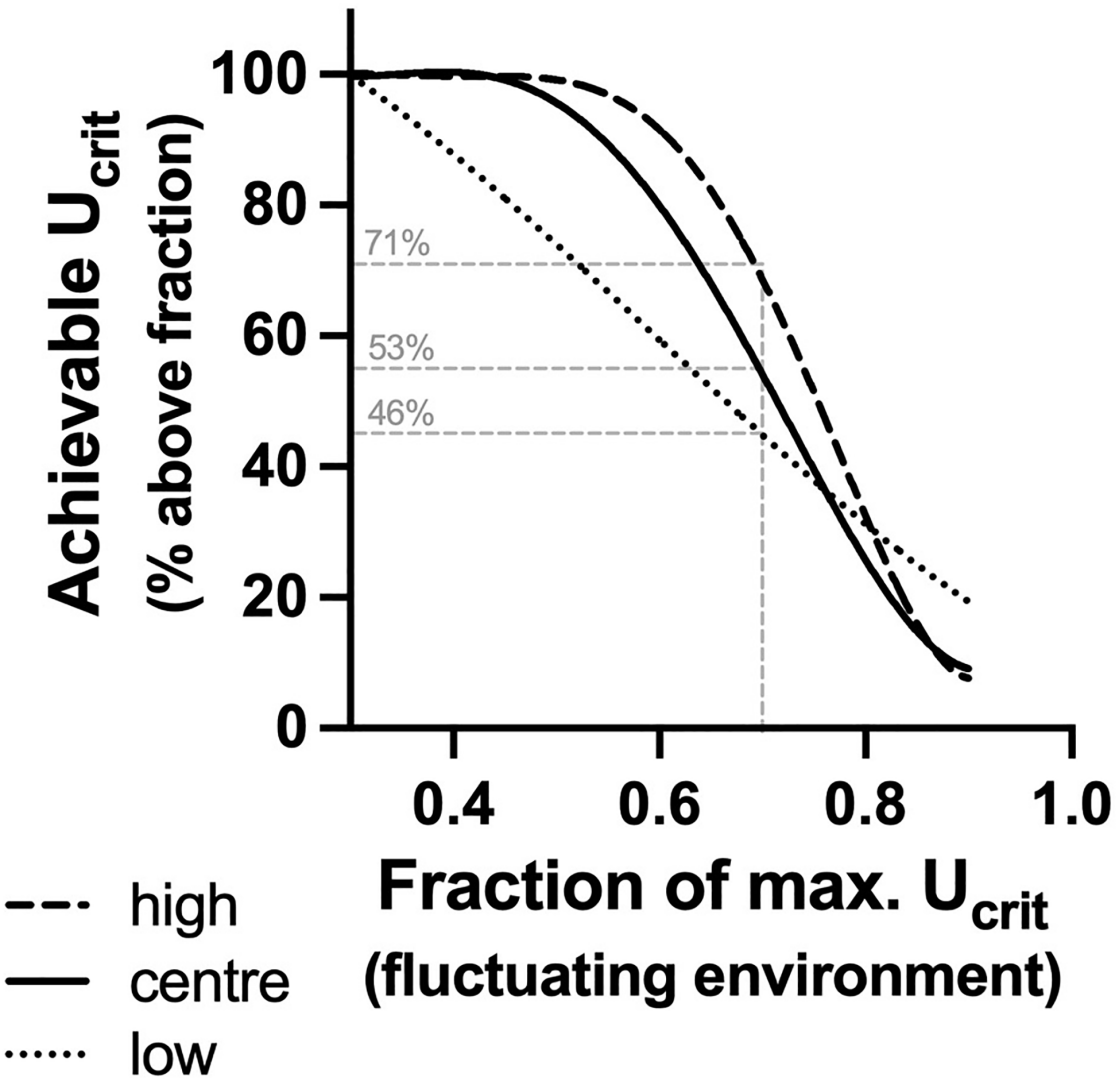
Achievable U_crit_ in a variable environment. Per cent of U_crit_ (= achievable U_crit_) in each subpopulation that was greater than a given fraction of the maximal (90th percentile) U_crit_ of the low acclimation capacity subpopulation. Fish with low acclimation capacity (low, fine broken line) had the highest maximum U_crit_ (90th percentile=11.7 BL s^−1^), but in a variable environment (20–28°C fluctuations), the achievable U_crit_ declined rapidly. In contrast, fish with central or high acclimation capacity [centre (solid line) and high (broken line), respectively] had lower maximum U_crit_ (90th percentile: 10.3 and 10.4 BL s^−1^, respectively), but their achievable U_crit_ was higher below approximately 0.8 of maximal U_crit_. The thin grey lines indicate a hypothetical scenario, where escape from a predator requires performance that is at least 0.7 of maximal U_crit_. Fish with high acclimation capacity would achieve this 71% of the time, while fish with low acclimation capacity would achieve this only relatively rarely (46%) with centre fish in between (53%).

The achievable U_crit_ in a variable environment was defined as the per cent of U_crit_ (= achievable U_crit_) that was greater than a given fraction of the maximal (90th percentile) U_crit_. In other words, the achievable U_crit_ is synonymous with the per cent of time fish could swim faster than a given fraction of maximum in an environment that varied over time (Figure 6). Fish with low acclimation capacity had the highest maximum U_crit_ (90th percentile=11.7 body lengths s^−1^), but in a variable environment, their achievable U_crit_ declined rapidly. This result is not surprising because in fish with low acclimation capacity, U_crit_ changes thermodynamically in proportion with the acute temperature change. In contrast, fish with central or high acclimation capacity compensated at least partially (centre) for the decline in environmental temperatures. Hence, even though these groups had lower maximum U_crit_ (90th percentile: 10.3 and 10.4 BL s^−1^, respectively), their achievable U_crit_ was higher than in the low acclimation subpopulation below approximately 0.8 of maximal U_crit_. The high acclimation capacity subpopulation had the highest achievable U_crit_ (Figure 6).

These nonlinear distributions of achievable U_crit_ could present a selective advantage for plastic phenotypes if the ecological outcomes of movement are maximised at a lower than maximal speed. Animals rarely move at their maximum speed, and the functional outcomes of responses such as escaping a predator may be optimised at fractions of maximal speed (Wilson et al., 2015). Success in escaping from a predator may be highest at a submaximal speed because at maximal speeds, precision of movement, information processing and endurance decline, while energetic costs increase (Wilson et al., 2015; Wynn et al., 2015). Conversely, speeds below a given threshold may simply be too slow for the prey to escape (Husak and Fox, 2006). Hypothetically, if it is assumed that a fraction of 0.7 maximal U_crit_ is necessary for escape from predators (Husak and Fox, 2006), the different distributions of achievable U_crit_ in our subpopulations will influence the success of escaping. Only 46% of U_crit_ values in the low acclimation capacity subpopulation fall above 0.7 maximal speed, while 71% of U_crit_ values were above 0.7 of maximal speed in the high acclimation capacity subpopulation. Hence, fish with high acclimation capacity would be vulnerable around 30% of the time, while those with low acclimation capacity would be vulnerable more than half the time. This hypothetical example demonstrates that the buffering of U_crit_ for low temperatures may translate to a selective advantage across acclimation conditions.

## Conclusion

Predictably variable environments are often thought to produce plastic phenotypes. Conversely, plasticity is thought to be selected against in stable environments, which implies that there is a cost of plasticity (DeWitt and Scheiner, 2004; Angilletta, 2009; Auld et al., 2010). Hence, the expectation is that depending on environmental context, populations – or even species – are comprised of either plastic or fixed phenotypes. We show that this is not the case, at least for mosquitofish. The trade-off between plasticity and maximal performance could be interpreted as a cost of plasticity because highly plastic individuals have lower maximal performance. However, animals rarely perform at maximal capacities under natural circumstances, and we show that plastic individuals have an advantage if the outcomes of fitness-related activities such as predator escape are optimised at submaximal performance levels.

Nonetheless, fixed phenotypes persist in the population, so that the advantages of plasticity are not sufficient to replace fixed phenotypes. It is possible that the greater performance of fixed individuals at warm temperatures may be advantageous during summer, when mean lake temperatures increase substantially and there are frequent heat waves. The capacity for plasticity may be outstripped by the degree of temperature rise during these times so that there is higher mortality of plastic phenotypes. Additionally, developmental temperatures can influence phenotypes, and cold conditions during early development produce more plastic individuals, and warm conditions produce individuals that perform better at warm temperatures (Seebacher et al., 2014; Loughland et al., 2021). In a short-lived species like mosquitofish, births at different times of year – and therefore at different temperatures – may suffice to balance population phenotypes. Trade-offs, developmental effects and the advantages of plastic phenotypes together are likely to explain the observed population variation. Note also that different traits within organisms can differ in the plasticity of their performance curves, which adds an additional layer of complexity and trade-offs (Wilson et al., 2002; Bozinovic et al., 2020). The contention that variable environments produce plasticity is likely to be too simplistic, because it does not capture the dynamics of natural populations.

## Data Availability Statement

Publicly available data sets were analysed in this study. These data can be found here: Electronic supplementary material for: Seebacher et al., 2015. Dryad Digital Repository: https://doi.org/10.5061/dryad.v6wwpzgs2

## Author Contributions

FS and AL conceived the ideas and edited the manuscript. FS analysed the data and wrote the manuscript. All authors contributed to the article and approved the submitted version.

## Funding

The research was funded by Australian Research Council Discovery grant DP190101168 to FS and AL.

## Conflict of Interest

The authors declare that the research was conducted in the absence of any commercial or financial relationships that could be construed as a potential conflict of interest.

## Publisher’s Note

All claims expressed in this article are solely those of the authors and do not necessarily represent those of their affiliated organizations, or those of the publisher, the editors and the reviewers. Any product that may be evaluated in this article, or claim that may be made by its manufacturer, is not guaranteed or endorsed by the publisher.
