# Supplementary material for: Plasticity of Performance Curves in Ectotherms: Individual Variation Modulates Population Responses to Environmental Change
Source: Front Physiol. 2021 Sep 28;12:733305. doi: 10.3389/fphys.2021.733305 (PMC8513571; doi:10.3389/fphys.2021.733305)
Supplement: Supplementary file 1 [file Data_Sheet_1.PDF]

```
#R code used in Seebacher and Little ms

#1 Subsampling
#take subsamples from the population ("Consequences of variation on
interpretation of samples")
#import data frame as csv file (or from Excel)
datum <- read.csv("file path.csv")
str(datum)
#specify number of iterations of loop to run
iterations =10
#specify output format
output <- list()
#run loop; number (8) refers to length of run (i.e. how many samples to
take per iteration)
for(i in 1:iterations){
  output[[i]] <- datum[sample(nrow(datum), 8),]
}
output
#Concatenate output so that variables w/ common names are merged into
rows from an array of lists (10 iterations=10 output lists)
datum2<-Map(c, output[[1]],
output[[2]],output[[3]],output[[4]],output[[5]],
output[[6]],output[[7]],output[[8]],output[[9]], output[[10]])
str(datum2)
#Convert to usable data frame
#Check that variables match up (20 variables/column names; 10 iterations
x 8 runs of loop =80 total data points)
df <- data.frame(datum2)
str(df)
#write these values to a file
write.csv(df, "file path.csv", row.names = FALSE)


#2 Model subpopulation responses
#("Consequences of individual variation for population responses")
#repeat calculation for random points in temperature cycle with a for
loop, and write it to a data frame

#set apmplitude and frequency for sinusoidal flucutations (note A=0 for
constant environment)
#dataset name here: highAC
highAC$A <- 4
highAC$f <- 0.1
#create empty data frame for results
result <- data.frame(matrix(NA, nrow = 61, ncol = 1))
#run loop containing functions 100x
for(i in 1:100){
  highAC$p <- runif(61, min=0, max=10)
  highAC$t <- runif(61, min =24, max = 24)+
(highAC$A*sin(highAC$f*highAC$time+highAC$p))
  result[[i]]<- highAC$P28+((28-highAC$t)*highAC$slope)

}
str(result)
#rename unwieldy column name
names(result)[names(result) == "matrix.NA..nrow...608..ncol...1."] <-
"V1"
str(result)
```

```
#combine columns
library(reshape2)
mod1<-melt(result)
mod2<-as.data.frame(mod1)
#variable refers to run of the loop; value is simulated data point
str(mod2)
#calculate CI
#formula: x ± z*(sd/sqrt(n))
#mean (x)
avg<-mean(mod2$value)
#standard devation (sd)
std.dev <- sd(mod2$value)
#z value for 95% CI= 1.96
CI<-1.96*(std.dev/sqrt(61))
#lower bound
lcl<-avg-CI
#upper bound
ucl<-avg+CI
#combine into data frame
final<-rbind(lcl,avg,ucl)
final
plot(final)
#write.csv(output, "file path", row.names = FALSE)

#get the number of values that are less than a given % of max Ucrit
(e.g., < 50% max); note that "<" is a logical operator so that
#the command counts the number of incidences that are "TRUE"
library(plyr)
#get value at 90th percentile
q <- quantile(mod2$value, probs = 0.9)

#calculate the number of values that are less than a given proportion of
the 90-th percentile
aa <- sum(mod2$value < q*0.1)
ab <- sum(mod2$value < q*0.2)
ac <- sum(mod2$value < q*0.3)
ad <- sum(mod2$value < q*0.4)
ae <- sum(mod2$value < q*0.45)
af <- sum(mod2$value < q*0.5)
ag <- sum(mod2$value < q*0.55)
ah <- sum(mod2$value < q*0.6)
ai <- sum(mod2$value < q*0.65)
aj <- sum(mod2$value < q*0.7)
ak <- sum(mod2$value < q*0.75)
al <- sum(mod2$value < q*0.8)
am <- sum(mod2$value < q*0.85)
an <- sum(mod2$value < q*0.9)
percent <- c(aa, ab, ac, ad, ae, af, ag, ah, ai, aj, ak, al, am, an)
percent
```
